# AGE-INDUCED SYSTEMIC REPROGRAMMING DRIVES DRUG RESISTANCE IN LUNG CANCER

**DOI:** 10.1093/geroni/igad104.0457

**Published:** 2023-12-21

**Authors:** Ana Gomes

**Affiliations:** Moffitt Cancer Center, Tampa, Florida, United States

## Abstract

Lung cancer accounts for the largest number of cancer-associated deaths in the United States. While great strides have been made due to the introduction of immunotherapies and targeted therapies against oncogenic drivers, chemotherapies remain the standard of care for the majority of lung cancer patients. However, many patients do not respond to these treatments or relapse following an initial response. We postulate that part of the problem is the lack of consideration in preclinical research and clinical trials of the main driver of lung tumorigenesis, the aging process. Here, we show that the organismal reprogramming that occurs with aging makes lung tumors in an aged host more conducive to withstand chemotherapies and targeted therapies. We traced this to an age-induced chronic accumulation of the stress hormone cortisol in circulation. Mechanistically our data show that chronic accumulation of cortisol drives the upregulation of metallothioneins—small, highly conserved, cysteine-rich metal-binding proteins—through activation of the glucocorticoid receptor in lung cancer cells, which in turn rewire these cells to be refractory to chemotherapies. Together, our work demonstrates a role for age-induced cortisol levels in promoting resistance to standard of care chemotherapies in lung cancer and offers a rationale for using therapeutic agents that block the glucocorticoid receptor to sensitize the average and most vulnerable lung cancer patients, the elderly, to chemotherapy.

